# Phagocytosis and Respiratory Burst Activity in Lumpsucker (*Cyclopterus lumpus* L.) Leucocytes Analysed by Flow Cytometry

**DOI:** 10.1371/journal.pone.0047909

**Published:** 2012-10-24

**Authors:** Gyri T. Haugland, Ragnhild Aakre Jakobsen, Nils Vestvik, Kristian Ulven, Lene Stokka, Heidrun I. Wergeland

**Affiliations:** 1 Department of Biology, University of Bergen, Bergen, Norway; 2 Aqua Kompetanse AS, Flatanger, Norway; University Medical Center Freiburg, Germany

## Abstract

In the present study, we have isolated leucocytes from peripheral blood, head kidney and spleen from lumpsucker (*Cyclopterus lumpus* L.), and performed functional studies like phagocytosis and respiratory burst, as well as morphological and cytochemical analyses. Different leucocytes were identified, such as lymphocytes, monocytes/macrophages and polymorphonuclear cells with bean shaped or bilobed nuclei. In addition, cells with similar morphology as described for dendritic cells in trout were abundant among the isolated leucocytes. Flow cytometry was successfully used for measuring phagocytosis and respiratory burst activity. The phagocytic capacity and ability were very high, and cells with different morphology in all three leucocyte preparations phagocytised beads rapidly. Due to lack of available cell markers, the identity of the phagocytic cells could not be determined. The potent non-specific phagocytosis was in accordance with a high number of cells positive for myeloperoxidase, an enzyme involved in oxygen-dependent killing mechanism present in phagocytic cells. Further, high respiratory burst activity was present in the leucocytes samples, verifying a potent oxygen- dependent degradation. At present, the specific antibody immune response could not be measured, as immunoglobulin or B-cells have not yet been isolated. Therefore, analyses of the specific immune response in this fish species await further clarification. The present study presents the first analyses of lumpsucker immunity and also the first within the order Scopaeniformes.

## Introduction

In teleosts, innate immunity is of vital importance as their adaptive immune system is considered to be less developed than in mammals. Phagocytosis, which is engulfment of particles, is an essential mechanism of the innate immune system and the first line of defence against invading pathogens in all eukaryotic organisms. In addition to macrophages, which are the main phagocytes in fish, fish B-cells and granulocytes have also potent phagocytic ability [1]–[11]. The three types of granulocytes; neutrophils, eosinophils and basophils, have been identified in fish, but their presence and morphology vary between fish species [12]–[15]. Further, due to confusion regarding granulocyte subset terminology and lack of cell specific surface markers, it is unclear which subtype who function as the main phagocytes in fish [16], [17], [18]. The functions of dendritic cells in fish are not yet known, as such cells have just recently been identified and isolated in a few fish species like salmon, zebrafish, medaka and trout [19]–[23].

Phagocytic cells are activated *in vivo* by a range of pathogen-associated molecular patterns, as well as by humoral components. *In vitro*, they are activated by various stimulants that also bind to their pattern recognition receptors [24]–[27]. However, for vertebrates and invertebrates a receptor independent activator, Phorbol 12-myristate 13-acetate (PMA), has been used in measurements of respiratory burst activity. We have earlier provided flow cytometry protocols for analyses of respiratory burst in cod and salmon using PMA as activator [28]. Respiratory burst is a potent oxygen-dependent killing mechanism in phagocytic cells, like monocytes/macrophages and neutrophils and is regarded as a highly efficient non-specific cellular defence mechanism. In some fish species, such non-specific mechanism might be crucial and provide the most significant immunity against pathogens. One example is cod that has low response of specific antibodies [29] and lack the gene encoding MHC II [30], [31]. However, cod might have other, yet unexplored mechanisms to provide specific protection.

In this study, we have performed morphological and functional studies of leucocytes isolated from lymphoid tissues and peripheral blood from lumpsucker. We have used a strategy based on flow cytometry to investigate functional mechanisms of innate immunity, which are possible without known genome sequence and cell markers. Such approaches might also be useful for others, who work with organisms where genome sequence is not yet available. The flow cytometry analyses provided accurate data from individual cells on phagocytic ability and capacity, and identified potent respiratory burst activity in lumpsucker leucocytes.

## Materials and Methods

The present work with lumpsucker has been conducted according to the approved national guidelines and performed according to prevailing animal welfare regulation. Rearing of fish under normal, optimal conditions does not require ethical approval under Norwegian law (FOR 1996-01-15 nr 23). All work in the presented manuscript has been done on cells isolated from dead fish. Fish were sacrificed with a sharp blow to the head, which is an appropriate procedure under Norwegian law.

**Figure 1:**
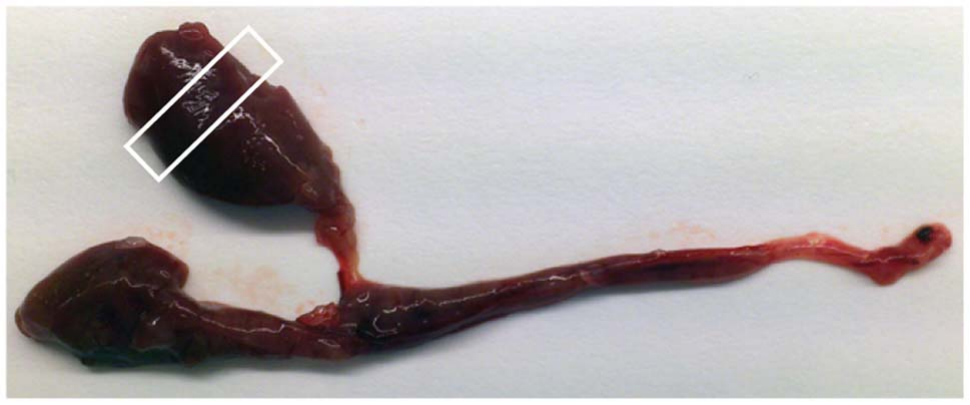
Lumpsucker kidney used for isolation of leucocytes. Dissected kidney where the section used for isolation of leucocytes from the left cranial lobe is marked.

### Fish

Lumpsucker (*Cyclopterus lumpus* L.) males, at a weight between 700 to 1000 g, from a group of wild caught fish intended for use as broodstock, were used. The fish was provided by Norsk Oppdrettservice in Flekkefjord, Norway. The fish (n = 40) were kept in two separate 500 l tanks in the rearing facilities at Bergen High-Technology Centre under normal optimal rearing conditions at a temperature of 6°C, salinity of 34 ‰ and 12∶12 hour light:dark. These facilities are approved by the Norwegian Animal Research Authority for rearing of fish. The water flow was 1000 l per hour and the fish were fed with the commercial dry feed Amber Neptune, marine feed for gadoid, obtained from Skretting, Norway. There were no signs of infection and no mortality in the fish.

**Figure 2:**
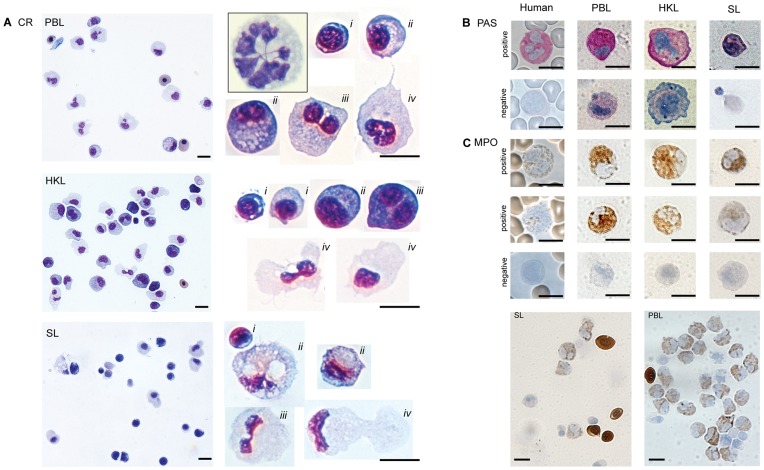
Morphological and cytochemical analyses of leucocytes isolated from peripheral blood, head kidney and spleen. Cytospin preparations of PBL, HKL, SL stained with Colorrapid (CR) (A), PAS (B) and MPO stained cells (C). The overview photos in A (left) and representative single cells (right), captured 630× magnifications. *i* = lymphocytes, *ii* = monocytes/macrophages, *iii* = polymorphonucear cells and *iv* = dendritic-like cells. The inset at top (right) in (A) show a polymorphonucleated cell (neutrophil) isolated from Atlantic salmon for comparison. In (B) and (C), representative single cells of isolated PBL, HKL and SL shown are captured at 630×. Representative cells from human blood smears are shown as controls (630×). Note that PAS staining (B) was highly variable and the two negative cells shown for PBL and HKL might be considered weak positive. In (C), overview of MPO stained SL and PBL, captured at 400×, show positive and negative leucocytes. Erythrocytes stain MPO positive. Scale bars = 5 µm.

### Sampling Procedure and Isolation of Leucocytes

Lumpsucker were randomly sampled for the experiments. The fish were quickly netted and killed by a sharp blow to the head. Peripheral blood (0.7 ml), collected from vena caudalis of the fish, was transferred to heparinised containers and diluted to a total volume of 5 ml in Leibovitz L-15+ (L-15 media without L-Glutamine adjusted to 370 mOsm by adding 5% (v/v) of a solution consisting of 0.41 M NaCl, 0.33 M NaHCO_3_ and 0.66% (w/v) D-glucose), supplemented with 100 µg ml^−1^ gentamicin sulphate (Lonza Biowhittaker Verviers, Belgium), 2 mM L-glutamine (Lonza Biowhittaker Verviers, Belgium), 10 U ml^−1^ heparin (Sigma-Aldrich, St. Louis, USA) and 15 mM HEPES (Sigma-Aldrich, St. Louis, USA)). Whole spleen was used for leucocyte isolation. The head kidney (HK) sample from lumpsucker was isolated from the left cranial HK lobe (Fig. 1). Tissue samples for leucocyte isolation were placed in 2 ml L-15+, and HK and spleen cell suspensions were obtained by gently forcing the tissue trough a cell strainer (Falcon, 100 µm (BD Biosciences Discovery Labware, Bedford, USA) using additional 3 ml L-15+. Leucocytes were isolated as previously described for cod [28]; the cell suspensions were placed on discontinuous Percoll gradients 3 ml 1.070 g ml^−1^ overlaid with 2.5 ml 1.050 g ml^−1^ and centrifuged 40 min at 400×g and 4°C. The leucocyte fraction was collected from the interface of the two Percoll densities including the downward density layer, and washed by diluting the suspension in L-15+ and centrifuged at 200×g for 10 min at 4°C. The cells were resuspended in 0.5 ml L-15+ and counted using a CASY Cell Counter™ (Innovatis AG, Mannheim, Germany). In addition, viability and aggregation factor for all isolated cell suspensions was determined using the CASY according to the manufacturer’s procedure. Leucocytes showed viability of 95% or above and the cell aggregation factor was below 2.0 for cell samples used in the analyses.

**Figure 3:**
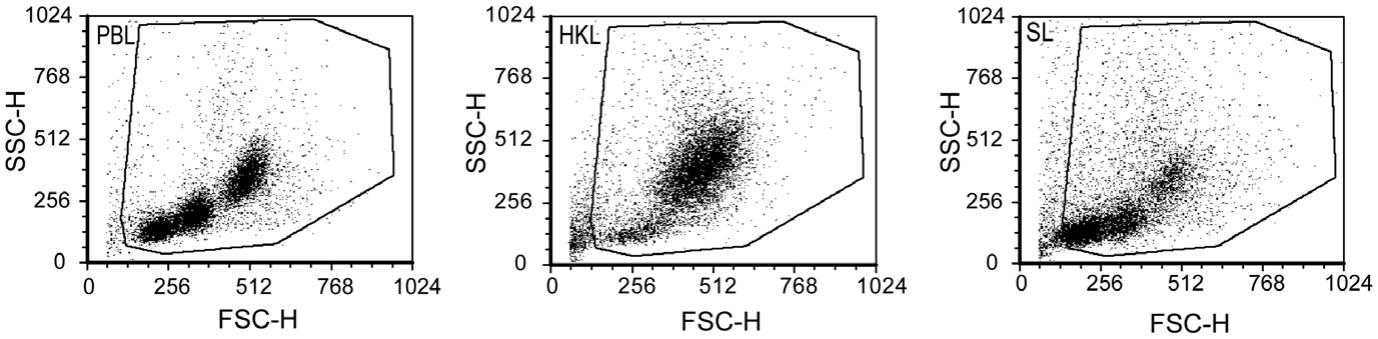
Flow cytometry analyses of leucocytes isolated from peripheral blood, head kidney and spleen. Representative size/granularity (FSC/SSC) dot plots, show different sub populations among PBL, HKL and SL. The regions used in the analyses, representing the live cells, are delimited in each panel.

### Cytospin Preparations

Cytospin preparations of isolated leucocytes were prepared by centrifugation of 100 µl of cell suspension of 1×10^6^ cells ml^−1^ at 1000 rpm, medium acceleration for 3 min, using a Shandon Cytospin III cytocentrifuge (Shandon Scientific Ltd, Runcorn, England). The cytospin preparations were air dried for 20 h at room temperature.

**Figure 4:**
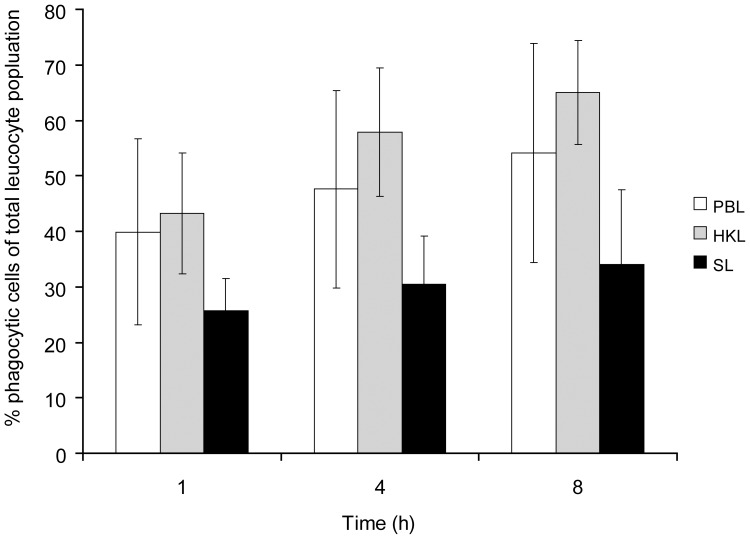
Isolated leucocytes have potent phagocytic ability. Proportions of phagocytic leucocytes of total PBL, HKL and SL after 1, 4 and 8 hours ingestion of fluorescent beads measured by flow cytometry (mean, bars indicate SD, N = 6).

**Figure 5:**
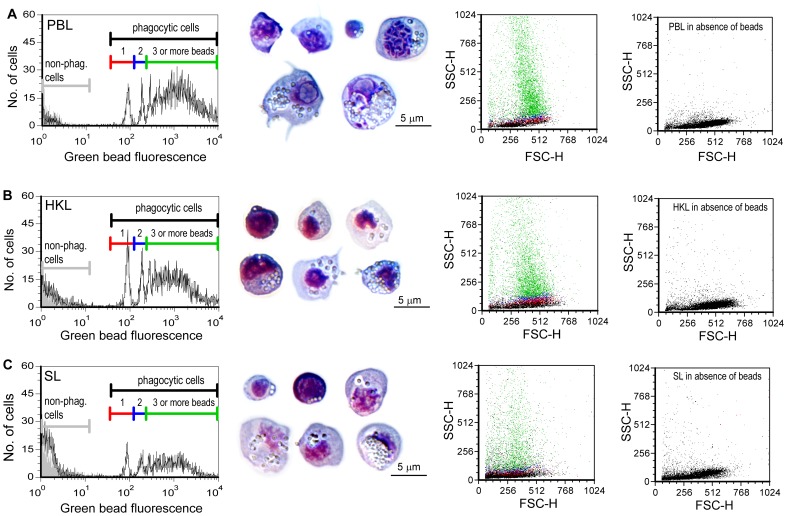
The phagocytic capacity of isolated leucocytes is high. FL1 (green bead fluorescence) histograms (left) showing phagocytic capacity of PBL (A), HKL (B) and SL (C) incubated with fluorescent beads (1 µm) for 4 h. Increased peak fluorescence indicates an increased number of ingested beads. Picture insets show cells stained with Colorrapid from PBL, HKL and SL samples that have ingested various numbers of beads. The left dot plots show cells in the red (cells with 1 bead) blue (cells with two beads) and green (cells with 3 or more beads) and black (non-phagocytic cells) regions; cells with a higher number of ingested beads have a higher granularity (SSC-value). The dot plots to the right show the light scatter properties of the cells incubated without beads at the instrument settings used for the phagocytosis assay.

### Cytochemistry

Cytochemical staining procedures were performed on isolated leucocytes in cytospin preparations as described above. Human control was own blood smear. Control salmon leucocytes for identification of neutrophil cells were isolated as described previously [32]. Cytospin preparations of cells were stained using Colorrapid-set from Lucerna-Chem (Lucerne, Switzerland).

**Figure 6:**
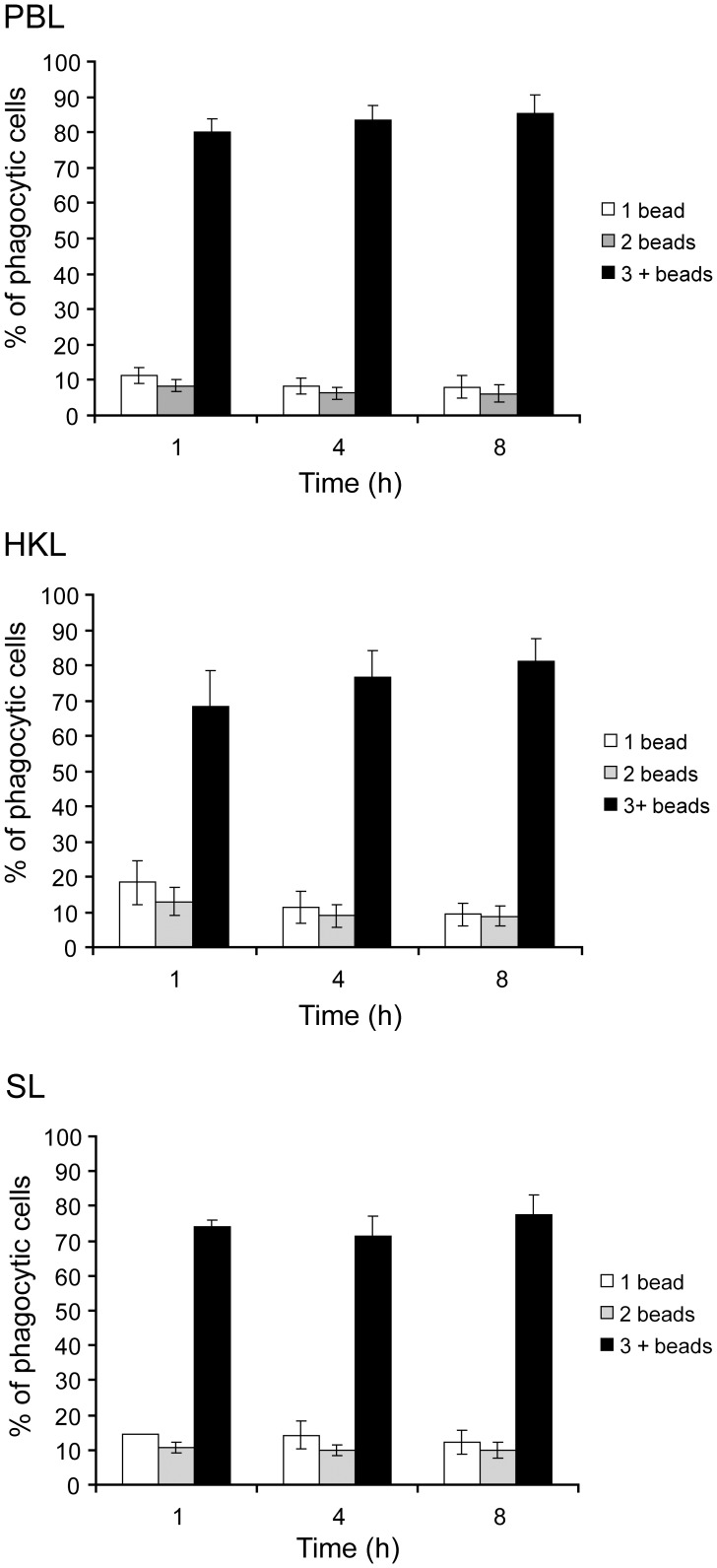
Phagocytic cells ingest beads rapidly. Proportions of phagocytic cells with various numbers of ingested beads in PBL, HKL and SL after incubation with fluorescent beads (1 µm) for 1, 4 or 8 h detected by flow cytometry (mean, bars indicate SD, N = 6 in all analysis except for SL 4 h where N = 5).

### Myeloperoxidase (MPO) Staining

Staining of cell preparations for MPO was done mainly as described by Ganassin *et al.* [33]. The preparations were incubated in freshly prepared fixative solution (10% (v/v) 37% formaldehyde, 90% (v/v) 95% ethanol) for 30 s, washed in gently running tap water and air dried in the dark for 10 min. The preparations were then incubated with diaminobenzidine (DAB) staining solution prepared from SIGMA*FAST* DAB tablets (Sigma-Aldrich, St. Louis, USA) for 30 min in the dark, rinsed in tap water and mounted in Dako Faramount Aqueous mounting medium (Dako, Carpinteria, USA). Staining was performed at room temperature.

**Figure 7:**
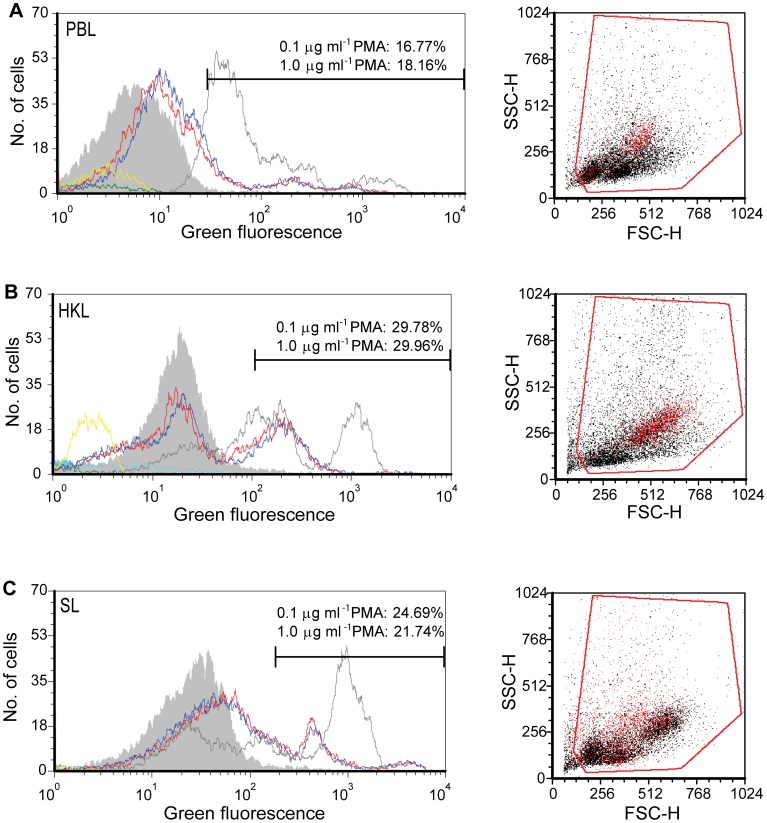
Isolated leucocytes show strong respiratory burst activity upon stimulation with PMA. Flow cytometry of respiratory burst in leucocytes from PBL (A), HKL (B) and SL (C). FL1 (green fluorescence) histograms show RHO fluorescence after PMA stimulation. Stimulated cells: 0.1 µg ml^−1^ of PMA, red line and 1 µg ml^−1^ of PMA, blue line. Controls: Non-stimulated cells without PMA and with DHR (grey filled peak), used for determination of limit between RHO positive and negative cells. Positive control for oxidation of DHR by H_2_O_2_ to RHO (grey line). Other negative controls: without PMA and DHR (aqua line), with PMA and without DHR (green line) and with H_2_O_2_, but without PMA and DHR (yellow line). These negative controls without DHR have low fluorescent intensity. Horisontal bars indicate RHO positive cells. The corresponding size/granularity (FCS/SSC) dot plots of PBL, HKL and SL show RHO positive cells (red) for PMA (1 µg ml^−1^) stimulated cells.

**Table 1:** The proportions of RHO-positive cells and geometric mean fluorescence intensity (GMFI) of PMA stimulated PBL, HKL and SL from lumpsucker analysed by flow cytometry.

	PBL^a^		HKL^b^		SL^a^	
PMA (µg ml^−1^)	% RHO-pos	GMFI	% RHO-pos	GMFI	% RHO-pos	GMFI
–	2.0	12.2±2.7	2.0	23.2±12.5	2.0	18.4±4.0
0.1	16.2±9.8	25.9±16.7	18.8±6.6	40.6±13.6	23.4±11.7	49.9±24.1
1.0	17.4±11.1	27.1±18.1	24.2±6.9	45.1±18.7	25.5±11.17	50.6±22.2

a N = 4, ^b^N = 5

### Periodic Acid Schiff (PAS) Staining

Staining was performed as described earlier for salmon cells [34]. Briefly, cell preparations were fixed in methanol for 10 min, rinsed in running tap water for 15 min and air dried. The preparations were then incubated with 0.044 M periodic acid for 10 min, rinsed in tap water and air dried, before incubation in Shiff’s reagent (Sigma-Aldrich, St. Louis, USA) for 10 min. After gentle rinsing in tap water, the preparations were counterstained for 20–30 min in filtered Mayer’s hematoxylin solution (Sigma-Aldrich, St.Louis, USA), rinsed in tap water and mounted in Dako Faramount Aqueous mounting medium (Dako, Carpinteria, USA).

**Figure 8:**
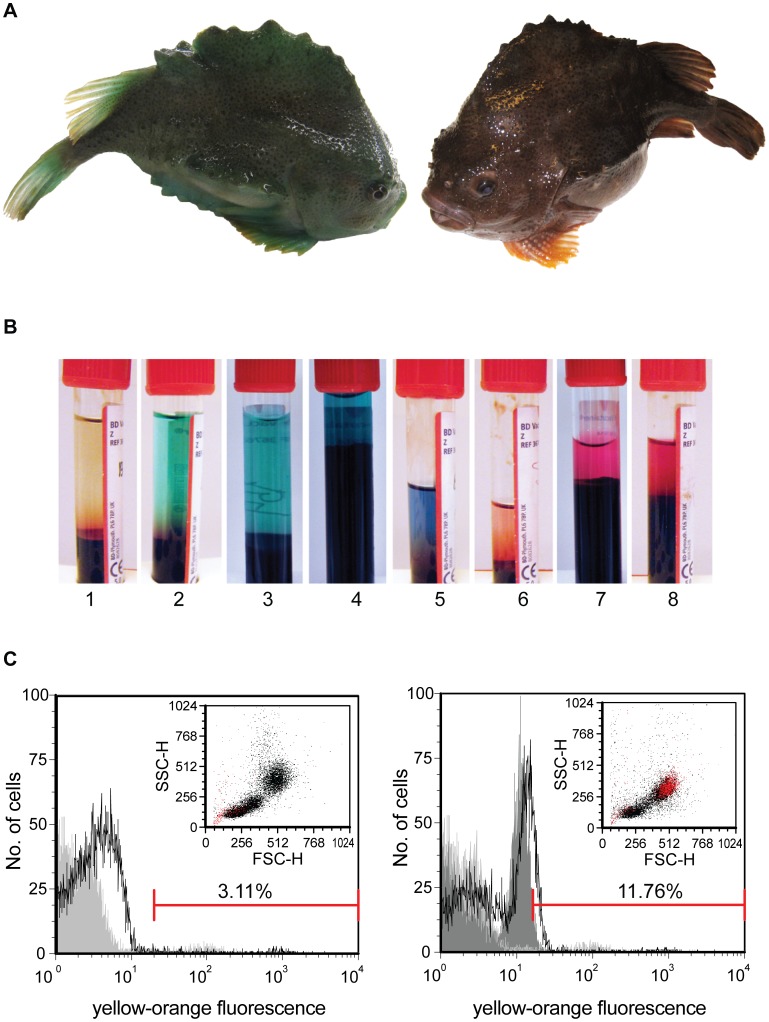
PBL samples from fish with purple/red serum give yellow-orange autofluorescence in flow analyses. The lumpsuckers varied in color from green to brown and red (A). Samples of serum from different fish (B). Histograms and dot plots (insets) when PI is added to leucocytes (C). In histogram: Gray without PI and black with PI. Horisontal bars show PI positive cells. This sample is used for gating of live cells to exclude dead cells from the analyses. The left figure in C shows PI positive cells (red dots) in dot plot for PBL from the green lumpsucker giving serum no 3, while the right figure shows the same results for the brown/red fish giving serum no 7. Note the difference in the yellow-orange fluorescence (red dots in C) for these two PBL samples.

### Flow Cytometry Assay of Phagocytosis

Phagocytosis was studied using fluorescent latex beads and flow cytometry as described earlier [7] with some modifications. Two hundred and fifty µl cell suspensions (1×10^7^ cells ml^−1^) were mixed with Fluoresbrite® YG carboxylate microspheres (Polysciences Inc., Warrington, USA), 1 µm in diameter, at a cell/bead ratio of 1∶25 per well in 48 well cell culture plates (Nunc, Roskilde, Denmark), and incubated for 1, 4 and 8 h at 12°C. Wells containing cell suspension without beads were used as negative controls. Following incubation with beads the cell suspensions were removed and the wells were washed twice, first with 250 µl L-15+ medium and thereby 250 µl PBS 380 mOsm (PBS 380). Adherent cells were loosened by trypsination for 5 min using 250 µl per well of trypsin-EDTA (Lonza Biowhittaker, Verviers, Belgium), and gentle scraping. To remove non–ingested beads, the cell suspension was placed on top of a cushion consisting of 3 ml phosphate-buffered saline (PBS), pH 7.3, with 3% (w/v) bovine serum albumin (Sigma, St. Louis, USA) and 4.5% (w/v) D-glucose, centrifuged at 100×g for 10 min at 4°C, washed once with 1 ml PBS+E (PBS containing 1% (w/v) BSA, 0.1% (w/v) sodium azide and 25 mM EDTA) and resuspended in 500 µl PBS+E prior to flow cytometry analyses.

Using flow cytometry, cells were analysed for forward scatter (FSC) and sideward scatter (SSC) patterns, representing the size and granularity of the cells, respectively, and for green bead fluorescence (detected with 530/30 nm bandpass filter; FL1). Dead cells were detected by staining the cells with 2 µg ml^−1^ propidium iodide (PI) (Sigma, St. Louis, USA) (using the 585/42 nm bandpass filter; FL2) and these cells were excluded from subsequent analyses by gating. The analyses were performed on a BD FACSCalibur flow cytometer (BD biosciences, San Jose, USA) equipped with a 15 mV 488 nm argon-ion laser using Cell Quest version 3.1 software (Becton Dickinson) recording 10 000 cells in each sample. Further data analyses were done using FCS express 3 (De Novo Software). Phagocytic ability was expressed as the percentage of total leucocytes with ingested beads, while the phagocytic capacity was expressed as the proportion of phagocytic cells that had ingested one, two, three or more beads.

### Flow Cytometry Assay of Respiratory Burst

The flow cytometry analyses of respiratory burst is based on previous established protocols for measurements of respiratory burst in cod and salmon where dihydrorhodamine 123 (DHR) is oxidised to the fluorescent rhodamine 123 (RHO) [28]. From a leucocyte concentration of 2.5×10^6^ ml^−1^, 200 µl was transferred to 5 ml polystyrene tube (Falcon, Becton Dickinson, Franklin lakes, USA) and incubated at 18°C for 10 min with gentle tilting. Respiratory burst was activated in leucocytes by PMA (Sigma, St. Louis, USA) using a concentration of 0.1 and 1 µg ml^−1^ PMA in the tube and incubated for 10 min at 18°C before addition of DHR. The PMA stock solution was made of 1 mg PMA in 1 ml DMSO (CH_3_)_2_SO, (Sigma, St. Louis, USA) and was stored at −20°C and further diluted in PBS 380 containing heparin (PBS 380 h). Five µl of 206 µM DHR, resulting in a total concentration of 5 µM per sample tube was added. The samples were mixed and incubated by gentle tilting for 15 min. Prior to flow cytometry analyses 300 µl PBS 380 h was added to each tube and the cells were carefully suspended using a vortex mixer. Two parallels per variable from 5 fish were used throughout all analyses.

The flow cytometry analyses and data analyses were performed as described above. RHO fluorescence was detected with 530/30 nm bandpass filter; FL1.

Several controls were included in the respiratory burst analyses. Untreated leucocytes control without PMA and DHR for detection of any possible auto fluorescence from the leucocytes. A PMA stimulated samples without DHR were analysed to test for presence of fluorescence not caused by oxidation of DHR. A non-stimulated sample added DHR was used to define the region of respiratory burst negative cells and this control was included throughout the experiments. A limit of 2% RHO positive non-stimulated leucocytes was defined. To verify that DHR was oxidized to RHO by H_2_O_2,_ which is produced in respiratory burst, H_2_O_2_ was added to leucocytes using 5 µl of 30% H_2_O_2_. Also, a H_2_O_2_ control without DHR was included. Samples were analysed with instrument settings both for non-treated leucocytes and for DHR treated leucocytes, and compared with cells from the same fish.

### Microscopy of Cells

Cytospin cell preparations and blood smears were examined using Zeiss Axioskop 2 plus microscope (Carl Zeiss, Germany). Cell suspensions prepared for analyses of respiratory burst activity in flow cytometry were studied by fluorescence microscopy. Cytospin preparations were made by centrifugation of 100 µl cell suspension for 3 min at 1000 rpm in a Shandon Cytospin III cytocentrifuge onto conventional glass slides. Preparations were immediately examined using fluorescence microscope Zeiss Axioskop 2 plus. Photos were prepared using Adobe photoshop CS5 (Adobe Systems Incorporated, San Jose, USA).

## Results

### Leucocyte Morphology and Enzyme Cytochemistry

Leucocytes from peripheral blood (PBL), head kidney (HKL) and spleen (SL) could be isolated, using the same densities of Percoll gradient as used for isolation of cod leucocytes. The leucocytes were present in one clear band at the interface between the two densities. In cytospin preparations of leucocytes, stained with Colorrapid (Fig. 2A), the cells appeared to be highly heterogeneous with respect to cell size and also the cell and nucleus morphology were highly variable. Lymphocytes, neutrophils and monocytes/macrophages were identified, and some of the cells contained many granules. Many large cells with irregular morphology and nucleus were abundant among PBL, HKL and SL. PAS positive cells were found among PBL and HKL, and a few clearly positive cells were also found among SL (Fig. 2B). The PAS reaction was highly variable, and many cells were weakly stained and could not easily be evaluated (Fig. 2B). When staining for MPO, numerous positive cells were found among PBL and HKL and the cells varied in shape and size as shown in Figure 2C. Also among SL, MPO positive cells were present, but not as many as among HKL and PBL (Fig. 2C).

### Flow Cytometry Revealed Cells with Different Sizes and Granularity

Flow cytometry analyses of PBL, HKL and SL confirmed heterogeneous cell populations. The distribution of cells in dot plots, presenting cell size versus granularity, is shown in Figure 3. Among PBL three clusters of cells were consistently observed, representing cells with different size and granularity, and all three clusters contained high numbers of cells. Among HKL large cells with high granularity were dominating and rather few small cells were present, while among SL small cells with low granularity were most frequent, although various sized larger cells with high and variable granularity were also found among the SL.

### Leucocytes Isolated from Blood, Spleen and Head Kidney have Potent Phagocytic Activity and Capacity

Potent phagocytic activity was observed for leucocytes both from blood, head kidney and spleen using flow cytometry (Fig. 4 and Fig. 5). The highest per cent phagocytic cells of the total number of leucocytes were measured in HKL for all time points; 1 hour (43.2%), 4 hours (57.9%) and 8 hours (65%). Among PBL, the percentages phagocytic cells were slightly lower, 39.9% (1 hour), 47.6% (4 hours) and 54.2% (8 hours), while leucocytes from spleen had lowest phagocytic activity with 25.8%, 30.4% and 34% phagocytic cells after 1, 4 and 8 hours, respectively.

Representative samples of PBL, HKL and SL incubated with fluorescent beads are shown in Figure 5. Potent phagocytic capacity, measured by number of beads each cell ingests, is shown in both histograms and scatter plots (Fig. 5). Cells containing beads varied in size, morphology and numerous beads ingested, as shown by microscopy of Colorrapid stained cytospin preparations of cells prepared for flow cytometry analyses. The phagocytic capacity over time, with the proportions of phagocytic cells containing one, two or more beads, are shown in Figure 6. Already after one hour exposure to beads, a high proportion of cells contain three or more beads, PBL (80.1%), HKL (68.4%) and SL (74.1%). From 1 to 8 hours there was only a slight increase, 3.3–5.5% in PBL and SL with 3 or more beads, while the proportion of cell in HKL increased by 12.9%. The proportions of cells with one or two beads were low, ranging in PBL, HKL and SL from 18.3% (HKL, 1 h) to 6.1% (PBL, 8 h). The highest percentages of cells with one and two beads were found after one hour and lowest after 8 h, and there was a slight decrease at 4 and 8 hours compared to 1 hour.

### Isolated Leucocytes have Strong Respiratory Burst Activity

PMA specifically stimulated respiratory burst activity in lumpsucker leucocytes. There was little difference in activity using 0.1 or 1 µg ml^−1^ PMA, and only slightly higher proportions of RHO positive cells were measured for 1 µg ml^−1^ as shown in Table 1 and Figure 7. The geometric mean fluorescence intensities (GMFI) of RHO positive cells are included in Table 1. Note that a fraction of leucocytes from blood and spleen had strong fluorescence intensity (Fig. 7), indicating high respiratory burst activity. Among PBL, both small cells (low FSC) with low granularity (low SSC), and larger cells with high granularity, had respiratory burst activity (Fig. 7). In HKL, RHO positive cells were typically large with high granularity, while positive cells isolated from spleen varied in size and granularity as shown in the dot plot (Fig. 7). The specificity of PMA for priming respiratory burst activity was confirmed by several controls included in the assay (Fig. 7). All negative controls had low fluorescent intensity, as shown in the histogram, while the positive control, containing H_2_O_2_, stimulated to high RHO production. Performing fluorescence microscopy of cytospun PMA stimulated cells, RHO positive cells appeared green, and the frequent occurrence among PBL, HKL and SL confirmed the results obtained using flow cytometry (data not shown).

### Autofluorescence in some of the PBL Samples

Interestingly, some PBL samples showed high yellow–orange autofluorescence, as shown in Figure 8C. This autofluorescence, observed in large cells with high granularity, was only found in fish with purple serum color from brown/red fish (Fig. 8A) providing sera no. 6–8 (Fig. 8B).

## Discussion

This is the first description of lumpsucker leucocytes and immune functions. Accordingly, no pre-existing protocols were available for isolation and functional studies. In this study, we have isolated lumpsucker leucocytes and performed functional studies of the essential non-specific immune mechanisms; phagocytosis and respiratory burst using flow cytometry.

The specific subtypes of leucocytes in lumpsucker could not easily be identified, including B-cells, as reagents for identification do not yet exist. However, based on morphological and cytochemical studies, using Colorrapid staining, PAS and MPO, various cell types were identified. Cytospin preparations revealed cells with heterogeneity in size and morphology among PBL, HKL and SL, and the cells were more similar to zebrafish, trout and cod leucocytes, than those in salmon. The most striking observation was large cells with irregular nuclei and cell morphology, similarly to enriched DCs from rainbow trout [20]. Also some very small leucocytes were observed among lumpsucker leucocytes, as found in cod [13]. The polymorphonucleated neutrophils are easily identified among the salmon leucocytes, but for lumpsucker, as for cod, this is not the case [13], [35], [36]. Some lumpsucker leucocytes with two lobes were, however, similar to neutrophils in zebrafish [23]. The neutrophils are granulocytes that are MPO positive and such cells have been verified in many fish species like more recently in Murray cod [37]. High proportions of cod and salmon neutrophils have been found both among PBL and HKL [7] and in lumpsucker numerous strongly MPO positive cells were present. Also among the SL, many MPO positive cells were observed, similarly to cod leucocytes [13]. The variable morphology of the MPO positive cells in lumpsucker can indicate different cell types, or different developmental stages as well as activation status of the cells. Other cells of the myeloid linage like monocytes can also be among the MPO positive cells. If lumpsucker B-cells are phagocytic and have oxygen-dependent killing capacities, which include myeloperoxidase, such cells can also be present as MPO positive small cells, but as most MPO cells seemed to be larger cells this might not be very likely. Cells that stained strongly positive for PAS were less frequent. PAS positive cells were observed among PBL, HKL and SL, but these could not easily be compared with MPO positive cells as the PAS staining intensity was highly variable and the discrimination between positive and negative cells was not clear. Normally there is an increase in polysaccharides during cell maturation, and the variations in PAS staining can therefore be ascribed to different maturation stages of cells. The neutrophils/heterophils are shown to be PAS positive in many fish species [37], [38], [39], [40] and one would expect that PAS positive cells were more frequently observed.

Myeloperoxidase production takes place in the phagolysosome, and is an important part of the anti-bacterial defence system of phagocytic cells. Therefore, the presence of numerous MPO positive cells indicated that professional phagocytic cells were present in the leucocyte preparations both from blood, head kidney and spleen. The results of the phagocytic ability and capacity analyses showed that, indeed, the phagocytic capacity of the cells to take up beads was very high. Almost maximum phagocytic capacity were obtained after one hour exposure to beads and at that time about 68 to 80% of the phagocytic cells had ingested three or more beads. The cytospin preparations of cells with ingested beads verified that cells of different sizes were phagocytic, both medium and large sized PBL, HKL and SL contained high numbers of beads. In addition, the diversity of the phagocytic cells was striking in all three cell preparations and the nature of the smallest cells is not known, being phagocytic they might belong to the myeloid linage. There was an increase in total phagocytic ability (mean values) from 1 to 8 hour in both PBL, HKL and SL and if one compare with similar analyses it is at least as high in PBL and HKL as for cod and higher than found in salmon [7].

The respiratory burst was measured using an assay where the oxidation of DHR by H_2_O_2_ is dependent on being catalysed by myeloperoxidase, cytochrome C or Fe^++^ [41]. H_2_O_2_ is regarded as a reliable component to quantify as it is the most stable oxygen reactive intermediate. PMA was found to activate the NADPH oxidase enzyme in lumpsucker cells and no negative effects on cell viability were observed as was seen for cod leucocytes when the highest concentration of 1 µg ml^−1^ was used. Thus, the concentration of PMA could be as used for activation of respiratory burst in salmon [28]. The RHO fluorescent cells were also easily in cytospin preparations as shown earlier for cod and salmon [28]. It is important that the fraction of dead cells is low after stimulation and in particular for lumpsucker, as in some fish, there was a high yellow-orange autofluorescence. We noticed that the autofluorescent was found in fish that had purple serum. The colour spectrum seen in sera from lumpsucker males has been observed and studied earlier [42]. While the blue/green colour has been described to biliverdin the component giving rise to the purple colour has not been identified, but it could be phycoerythrin [43], [44]. The absorbance of the various pigments in lumpsuker serum has been studied and the red pigment in male serum had a peak at 536 nm [44] which is in the absorbance range of PI, and also the emission ranges are overlapping. It was therefore particularly important for the flow cytometry analyses in these fish that few cells died due to PMA stimulation, or during the respiratory burst assay. Since proportion of dead cells was low in PBL in the present study there was no problem with the gating of cells in respiratory burst assay as can be seen from the dot plots of PI treated PBL. The various chromophores present in samples from lumpsucker might cause problems with autofluorescence in assays, for example using fluoro marked reagents like phycoerythrin. The chromophore registered in this study can be normally present in the cells, or present due to phagocytised material in phagocytic cells. Being detected in larger and more granulated cells and since no autofluorescence were found among HKL and SL, phagocytosis seems plausible.

The percentages of RHO positive cells in PBL and HKL were in the range observed for cod and salmon [28]. In the present study, respiratory burst was also analysed for spleen cells and these had the highest percentage of positive cells and GMFI values. Individual variations among SL, as among PBL and HKL, are similar to measurements in cod and salmon. Presently, fish immunology studies include the species Atlantic salmon and rainbow trout (order Salmoniformes), zebrafish (order Cypriniformes), channel catfish (order Siluroformes), Atlantic cod (order Gadidae), medaka (order Beloniformes), seabass, seabeam and Murray cod (order Perciformes) and fugu (order Tetraodontiformes). These studies have revealed that considerable differences in the immune components are present within the teleosts, such as lack of MHCII in cod [30], [31]. As such, this study of immune cells in lumpsucker (order Scopaeniformes), contribute to broaden our knowledge of immunity in teleosts. In addition, lumpsucker is becoming important for the aquaculture industry as it is now used as cleaner fish for sea lice on Atlantic salmon. However, farming of this species is challenging as it is susceptible to infections itself, and therefore knowledge of its immunity is required. Our findings of high activity of the most important non-specific defence mechanisms, phagocytosis and respiratory burst, show that immunostimulation of these mechanisms is crucial in early rearing stages to prevent bacterial infections. As such, the first steps in antigen presentation route are highly active, but it remains to see if the specific antibody response and MHC pattern resembles that of cod resulting in low specific antibody levels after stimulation [30] or if the lumpsuckers have good humoral immune response such as salmon. Thus, further exploration of adaptive immunity in lumpsucker and the effects of vaccines await further clarification.

## References

[1] Chaves-Pozo E, Mulero V, Meseguer J, Garcia Ayala A (2005) Professional phagocytic granulocytes of the bony fish gilthead seabream display functional adaptation to testicular microenvironment. J Leukoc Biol 78: 345–351. 15937143 10.1189/jlb.0205120

[2] do Vale A, Afonso A, Silva MT (2002) The professional phagocytes of sea bass (*Dicentrarchus labrax* L.): cytochemical characterisation of neutrophils and macrophages in the normal and inflamed peritoneal cavity. Fish Shellfish Immunol 13: 183–198. 12365730 10.1006/fsim.2001.0394

[3] Esteban MA, Meseguer J (1997) Factors influencing phagocytic response of macrophages from the sea bass (*Dicentrarchus labrax* L.): an ultrastructural and quantitative study. Anat Rec 248: 533–541. 9268142 10.1002/(SICI)1097-0185(199708)248:4<533::AID-AR5>3.0.CO;2-M

[4] Esteban MA, Mulero V, Munoz J, Meseguer J (1998) Methodological aspects of assessing phagocytosis of *Vibrio anguillarum* by leucocytes of gilthead seabream (*Sparus aurata* L.) by flow cytometry and electron microscopy. Cell Tissue Res 293: 133–141. 9634605 10.1007/s004410051105

[5] Lamas J, Ellis AE (1994) Atlantic salmon (*Salmo salar*) neutrophil responses to Aeromonas salmonicida. Fish Shellfish Immunol 4: 201–219.

[6] Li J, Barreda DR, Zhang YA, Boshra H, Gelman AE, et al (2006) B lymphocytes from early vertebrates have potent phagocytic and microbicidal abilities. Nat Immunol 7: 1116–1124. 16980980 10.1038/ni1389

[7] Øverland HS, Pettersen EF, Ronneseth A, Wergeland HI (2010) Phagocytosis by B-cells and neutrophils in Atlantic salmon (*Salmo salar* L.) and Atlantic cod (*Gadus morhua* L.). Fish Shellfish Immunol 28: 193–204. 19874896 10.1016/j.fsi.2009.10.021

[8] Secombes CJ (1996) The nonspecific immune system: cellular defenses. In: Iwama G, Nakanishi T, editors. The fish immune system. Organism, pathogen and environment. San Diego: Academic Press. 63–95.

[9] Sepulcre MP, Lopez-Castejon G, Meseguer J, Mulero V (2007) The activation of gilthead seabream professional phagocytes by different PAMPs underlines the behavioural diversity of the main innate immune cells of bony fish. Mol Immunol 44: 2009–2016. 17087994 10.1016/j.molimm.2006.09.022

[10] Steiro KAJ, Gildberg AJB (1998) Optimising of culture conditions and stimulation of head kidney macrophages from Altantic cod, *Gadus morhua* L. J Fish Dis. 21: 335–344.

[11] Sørensen KK, Sveinbjornsson B, Dalmo RA, Smedsrod B, Bertheussen K (1997) Isolation, cultivation and characterization of head kidney macrophages from Atlantic cod, *Gadus morhus* L. J Fish Dis. 20: 93–107.

[12] Ainsworth WJ (1992) Fish granulocytes: Morphology, distribution and function. Annu Rev Fish Dis 2: 123–148.

[13] Ronneseth A, Wergeland HI, Pettersen EF (2007) Neutrophils and B-cells in Atlantic cod (*Gadus morhua* L.). Fish Shellfish Immunol 23: 493–503. 17475507 10.1016/j.fsi.2006.08.017

[14] Rowley A, Hunt T, Pettersen E, Mainwaring G (1988) Fish. In: Rowley ARN, editor. Vertebrate blood cells. Cambridge: University Press. 19–127.

[15] Zapata AG, Chibá A, Varas A (1996) Cells and tissue of the immune system of fish. In: Iwama G, Najanishi T, editors. The fish immune system. London: Academic Press 1–53.

[16] Crowhurst MO, Layton JE, Lieschke GJ (2002) Developmental biology of zebrafish myeloid cells. Int J Dev Biol 46: 483–492. 12141435

[17] Lieschke GJ, Oates AC, Crowhurst MO, Ward AC, Layton JE (2001) Morphologic and functional characterization of granulocytes and macrophages in embryonic and adult zebrafish. Blood 98: 3087–3096. 11698295 10.1182/blood.v98.10.3087

[18] Sepulcre MP, Pelegrin P, Mulero V, Meseguer J (2002) Characterisation of gilthead seabream acidophilic granulocytes by a monoclonal antibody unequivocally points to their involvement in fish phagocytic response. Cell Tissue Res 308: 97–102. 12012209 10.1007/s00441-002-0531-1

[19] Aghaallaei N, Bajoghli B, Schwarz H, Schorpp M, Boehm T (2010) Characterization of mononuclear phagocytic cells in medaka fish transgenic for a cxcr3a:gfp reporter. Proc Natl Acad Sci U S A 107: 18079–18084. 20921403 10.1073/pnas.1000467107 PMC2964234

[20] Bassity E, Clark TG (2012) Functional identification of dendritic cells in the teleost model, rainbow trout (*Oncorhynchus mykiss*). PLoS One 7: e33196. 22427987 10.1371/journal.pone.0033196 PMC3299753

[21] Haugland GT, Pettersen EF, Sviland C, Ronneseth A, Wergeland HI (2010) Immunostaining of Atlantic salmon (*Salmo salar* L.) leucocytes. J Immunol Methods 362: 10–21. 20674576 10.1016/j.jim.2010.07.008

[22] Lovy J, Wright GM, Speare DJ (2006) Morphological presentation of a dendritic-like cell within the gills of chinook salmon infected with *Loma salmonae* . Dev Comp Immunol 30: 259–263. 16139356 10.1016/j.dci.2005.06.003

[23] Lugo-Villarino G, Balla KM, Stachura DL, Banuelos K, Werneck MB, et al (2010) Identification of dendritic antigen-presenting cells in the zebrafish. Proc Natl Acad Sci U S A 107: 15850–15855. 20733076 10.1073/pnas.1000494107 PMC2936643

[24] Alvarez-Pellitero P (2008) Fish immunity and parasite infections: from innate immunity to immunoprophylactic prospects. Vet Immunol Immunopathol 126: 171–198. 18783835 10.1016/j.vetimm.2008.07.013

[25] Connor MA, Jaso-Friedmann L, Leary JH III, Evans DL (2009) Role of nonspecific cytotoxic cells in bacterial resistance: expression of a novel pattern recognition receptor with antimicrobial activity. Mol Immunol 46: 953–961. 19007992 10.1016/j.molimm.2008.09.025

[26] Magnadottir B (2006) Innate immunity of fish (overview). Fish Shellfish Immunol 20: 137–151. 15950491 10.1016/j.fsi.2004.09.006

[27] Pasare C, Medzhitov R (2004) Toll-like receptors and acquired immunity. Semin Immunol 16: 23–26. 14751760 10.1016/j.smim.2003.10.006

[28] Kalgraff CA, Wergeland HI, Pettersen EF (2011) Flow cytometry assays of respiratory burst in Atlantic salmon (*Salmo salar* L.) and in Atlantic cod (*Gadus morhua* L.) leucocytes. Fish Shellfish Immunol 31: 381–388. 21672631 10.1016/j.fsi.2011.05.028

[29] Magnadottir B, Jonsdottir H, Helgason S, Bjornsson B, Solem ST, et al (2001) Immune parameters of immunised cod (*Gadus morhua* L.). Fish Shellfish Immunol 11: 75–89. 11271604 10.1006/fsim.2000.0296

[30] Pilstrom L, Warr GW, Stromberg S (2005) Why is the antibody response of Atlantic cod so poor? The search for a genetic explanation. Fisheries Sci 71: 961–971.

[31] Star B, Nederbragt AJ, Jentoft S, Grimholt U, Malmstrom M, et al (2011) The genome sequence of Atlantic cod reveals a unique immune system. Nature 477: 207–210. 21832995 10.1038/nature10342 PMC3537168

[32] Pettersen EF, Fyllingen I, Kavlie A, Maaseide NP, Glette J, et al (1995) Monoclonal antibodies reactive with serum IgM and leukocytes from Atlantic salmon (*Salmo salar* L). Fish Shellfish Immunol 5: 275–287.

[33] Ganassin RC, Schirmer K, Bols M (2000) Cell and tissue culture; In: Ostrander GK, editor. The laboratory fish. London: Academic Press. 631–651.

[34] Pettersen EF, Ingerslev HC, Stavang V, Egenberg M, Wergeland HI (2008) A highly phagocytic cell line TO from Atlantic salmon is CD83 positive and M-CSFR negative, indicating a dendritic-like cell type. Fish Shellfish Immunol 25: 809–819. 18817880 10.1016/j.fsi.2008.08.014

[35] Pettersen EF, Bjerknes R, Wergeland HI (2000) Studies of Atlantic salmon (*Salmo salar* L.) blood, spleen and head kidney leucocytes using specific monoclonal antibodies, immunohistochemistry and flow cytometry. Fish Shellfish Immunol 10: 695–710. 11185754 10.1006/fsim.2000.0284

[36] Zinkl JG, Cox WT, Kono CS (1991) Morphology and cytochemistry of leucocytes and thrombocytes of six species of fish. Comp Haematol Int 1: 187–195.

[37] Shigdar S, Harford A, Ward AC (2009) Cytochemical characterisation of the leucocytes and thrombocytes from Murray cod (*Maccullochella peelii peelii*, Mitchell). Fish Shellfish Immunol 26: 731–736. 19332132 10.1016/j.fsi.2009.03.010

[38] Burrows AS, Fletcher TC, Manning MJ (2001) Haematology of the turbot, *Psetta maxima* (L.): ultrastructural, cytochemical and morphological properties of peripheral blood leucocytes. J Applied Ichthyol 17: 77–84.

[39] Garavini C, Martelli P, Borelli B (1981) Alkaline phosphatase and peroxidase in neutrophils of the catfish *Ictalurus melas (Rafinesque) (Siluriformes Ictaluridae*). Histochemistry 72: 75–81. 6169697 10.1007/BF00496781

[40] Meseguer J, Lopez-Ruiz A, Angeles Esteban M (1994) Cytochemical characterization of leucocytes from the seawater teleost, gilthead seabream (*Sparus aurata* L.). Histochemistry 102: 37–44. 7814268 10.1007/BF00271047

[41] Mohanty JG, Jaffe JS, Schulman ES, Raible DG (1997) A highly sensitive fluorescent micro-assay of H_2_O_2_ release from activated human leukocytes using a dihydroxyphenoxazine derivative. J Immunol Methods 202: 133–141. 9107302 10.1016/s0022-1759(96)00244-x

[42] Davenport J, Thorsteinsson V (1989) Observation on the colors of lumpsuckers, *Cyclopterus lumpus* L. J Fish Biol. 35: 829–838.

[43] Fang LS, Bada JL (1990) The blue-green blood plasma of marine fish. Comp Biochem Physiol B 97: 37–45. 2253479 10.1016/0305-0491(90)90174-r

[44] Mudge SM, Davenport J (1986) Serum pigmentation in *Cyclopterus lumpus* L. J Fish Biol. 29: 737–745.

